# Biological Effect of Di (p-methylbenzoyl) Diselenide (*In*-*vitro*) and Its Acute Hepatotoxicity on Rats (*In*-*vivo*) 

**Published:** 2014

**Authors:** Galila Ahmed Yacout, Doaa Ahmad Ghareeb, Shaymaa Ali El-hamshary, Mohamed Mohamed El-Sadek

**Affiliations:** a *Biochemistry Department, Faculty of Science, Alexandria University, Alexandria, Egypt. *; b *Chemistry Department, Faculty of Science, Alexandria University, Alexandria, Egypt. *

**Keywords:** Diselenide, Oxidative stress, Arginase

## Abstract

Selenium plays an important role in biological system due to its incorporation in glutathione peroxidases and thioredoxin reductase as prosthetic group, the pharmacological studies of synthetic organoseleno-compounds revealed these molecules to be used as antioxidants, enzyme inhibitors, neuroprotectors, antitumor, anti-infectious agents, cytokine inducers and immuno-modulators. The present study was undertaken to elucidate Synthesis and biological effect Di (p-methylbenzoyl) diselenide (DMBDS) *in-vitro*. Di (*p*-methylbenzoyl) diselenide DMBDS was synthesized and its structure was confirmed by different spectroscopy techniques. *In-vitro *dose response of DMBDS on lipid peroxidation, nitrite content, GPx and arginase activities beside blood coagulation were measured. Acute toxicological effects were assessed by single orally injected Swiss albino mice with different DMBDS concentrations. *In-vitro *results revealed that DMBDS induces oxidative stress, elevation of arginase activity and acts as coagulant.

## Introduction

Selenium plays an important role in biological system due to its incorporation in glutathione peroxidases and thioredoxin reductase as prosthetic group, it is accounted as a potent antioxidant (1). Meanwhile, toxicity of selenium returns to its ability to oxidize the protein and non- protein thiol groups, this oxidation process simultaneously generates the superoxide molecules that damage cellular components (2). Therefore, selenium can be acting as prooxidants or antioxidants. 

However, numerous studies have established that selenium methionine and selenium-enriched yeast are the most appropriate forms of selenium as nutritional supplements due to their excellent bioavailability and lower toxicity among various selenium forms (3). 

In the last few decades, the pharmacological studies of synthetic organoseleno-compounds revealed these molecules to be used as antioxidants, enzyme inhibitors, neuroprotectors, antitumor, anti-infectious agents, cytokine inducers and immuno-modulators (4). Li and Cao (5) reported that synthetic organoseleno-compounds are potent antioxidants due to the strong ability to scavenge intracellular reactive oxygen species (ROS) and prevent oxidative damage, so they could be used as DNA protectors, cellular protectors and pre-neoplastic changes inhibitors that took place ddue to aflatoxin B1 cytotoxicity (6). Moreover, diselenides are good candidates to become therapeutic antioxidants because they exert glutathione peroxidase-like activity via reacting with (-SH) groups (7). Diphenyl diselenide is a simple and stable organoseleno-compound, acts as electrophilic reagent used in the synthesis of a variety of pharmacologically active compounds and has antioxidant, hepatoprotective, neuroprotective, anti-inflammatory, and hypolipidemic effect (4-8). Also diphenyl diselenide is less toxic than other seleno-compounds (9-10).

Chemical toxicity *in-vivo *is ultimately a process that occurs at the cellular level and thus can be studied *in-vitro*. The ability to obtain an approximate prediction of the *in-vivo *toxic dose by means of a simple *in-vitro *test could reduce the suffering caused during *in-vivo *testing by permitting the selection of selenium doses which would be better tolerated by the animals and this might, in turn, also reduce the overall number of animals used.

Pur study aimed to synthesize and investigate the *in-vitro *biological and/or toxicological activity of new organoseleno-compounds di(p-methylbenzoyl) diselenide (DMBDS).

## Experimental

*Chemicals and reagents*

Glutathione, dithiobisnitrobenzoic acid (DTNB), cumene hydroperoxide, absolute ethanol, sodium borohydride (98%), n-hexane and dichloromethane were purchased from Sigma-Aldrich, USA. Molecular sieves type 4A°, potassium bromide, *p*-methylbenzoyl chloride and absolute methanol were purchased from Fluka, USA. Ethylene glycol (99%) and calcium chloride dihydrate were purchased from Aldrich Chemical Company *Ltd*., England. Black selenium powder was purchased from Oxford Laboratory, India. Tris-hydrochloride and trichloroacetic acid were purchased from Loba Chemie Pvt. *Ltd*., India. Diethyl ether was purchased from Nice Chemicals Pvt. *Ltd*., India. Thromboplastin reagent (PT) was purchased from Futura system, Roma, while partial thromboplastin time reagent (APTT) and prothrombin time reagent (PT) was purchased from BioSTC, Roma. 

*Synthesis of di(p-methylbenzoyl)diselenide (* 11 *)*

Black selenium powder (8 g) was suspended in absolute ethanol (100 mL) for 15 min at 0 ºC then sodium borohydride (4.5 g) were added. Through one hour on shaker, *p–*methylbenzoyl chloride (13.2 mL) was slowly added with stirring at 0 ºC then ethanolic mixture of iodine and potassium iodide was added. The obtained mixture was extracted three times with dichloromethane. The collected dichloromethane layer was washed with sodium bicarbonate solution (1 %), followed by distilled water. The organic layer was dried over anhydrous sodium sulphate, filtered and concentrated using rotary evaporator. The obtained diselenide (an orange crystalline solid) was filtered off and dried (17.0 g).

*Atomic absorption spectra of DMBDS (* 11 *)*

DMBDS (0.002-0.003 g) was mixed with concentrated nitric acid (8 mL) and evaporated till dryness. After cooling, the residue was diluted with 50 mL distilled water, and then subjected to atomic absorption spectrophotometer “Perkin-Elmer” 2380, JEOL JNM ECA, 500 MHZ.

*Infra red spectra of DMBDS (* 11 *)*

A disc of DMBDS (1 mg) and potassium bromide (100 mg) was formed and examined by Infra-red system “Perkin-Elmer” Spectrum RX IFT-IR System.

*NMR spectra of DMBDS (* 11 *)*

Sample of DMBDS (20 mg) was dissolved in chloroform (0.7 mL, deuterium) and about 0.38-0.42 mL of DMBDS solution was examined using JEOL JNM ECA, 500 MHZ, NMR-spectrophotometer.

*Biological activity of dmbds*

*Preparation of liver homogenate*

Six albino mice-livers were individually homogenized according to Lin *et al*., (12), with 9 volumes of phosphate buffer (0.1 M, pH 7.4) using Tekmar homogenizer. The obtained solution was centrifuged off at 3000 rpm for 15 minutes at 4 °C and the collected supernatant was stored at – 20 ºC until used.

*Preparation of stock solution of DMBDS*

Stock solution of DMBDS (100 μM) was prepared in chloroform, then kept in a dark bottle and stored at +8 ºC until used.

*Effect of DMBDS on GPx, arginase, activities and nitrite content*

In separate test tubes, 1 mL of liver homogenate was mixed with 1 mL of DMBDS with different concentrations (0, 1, 5, 10, 50 and 100 μM) then incubated at 37 ºC for 45 min, and then nitrite content (13), GPx (14) and arginase (15) activities were measured, respectively.

*Effect of DMBDS on lipid peroxidation*

Different concentrations of DMBDS (2 mL each), were incubated with equal volume of liver homogenate at 37 ºC for about 45 min. Blank experiment was carried out as above mentioned using chloroform instead of DMBDS. *In-vitro *tissue lipid peroxidation was induced by adding H_2_O_2_ and ferrous sulphate to final concentrations of 1 mM and 0.5 mM, respectively, for both test and control reaction mixtures (Ref.). After an incubation period of about 180 min at 37 ºC, butylated hydroxyl toluene at (final concentration 0.02%) was added to stop the peroxidation. The obtained mixture was centrifuged at 3000 rpm for 15 min, then 1 mL of the resultant supernatant was mixed with 1 mL of TCA (15%) followed by centrifugation at 3000 rpm for 10 min. The resultant TBARS was assayed according to Wills (16).

*Effect of DMBDS on human plasma activated partial thromboplastin time (APTT) and prothrombin time (PT)*

Normal human blood sample (9 mL) was collected by venipuncture and drawn into 1 mL of 3.8% sodium citrate. Blood was centrifuged at 3000 rpm for 15 min and the plasma was collected. Citrated normal human plasma (100 μL) was mixed with 100 μL of different DMBDS concentrations (0, 0.5 and 1.1 mg/mL) and incubated for 5 min at 37 ºC (17). 

For PT measurement, 100 μL PT reagent was added and clotting time was recorded, meanwhile for APTT measure, 100 μL APTT reagent was added then incubated for further 5 min at 37 ºC followed by adding 100 μL CaCl2 and clotting time was recorded (17).

*Animals*

Swiss albino mice aged two months were obtained from the animal house of Faculty of Medicine, Alexandria University, Alexandria, Egypt, housed in standard cages where food and water were provided *ad- libitum. *

*LD 50 measurement*

Different doses of DMBDS were prepared started from 1 μg till 10 g/Kg/mL corn oil. Each dose was orally administrated to a group of Swiss albino mice (6 mice/ group) for 14 days. All treated animals were still alive along the experimental period. 

*Statistical analyses*

The obtained data expressed as mean ± S.D. One-way analysis of variance (ANOVA) followed by Student Newman-Keuls test, which was provided by Primer Biostatistics program (Version 5).

## Results

Properties of DMBDS in Table 1 showed the chemical structure as C_16 _H_14_ O_2_ Se_2_, molecular weight of 396.201 g/mol and melting point of 176 ºC. 

**Figure 1:**
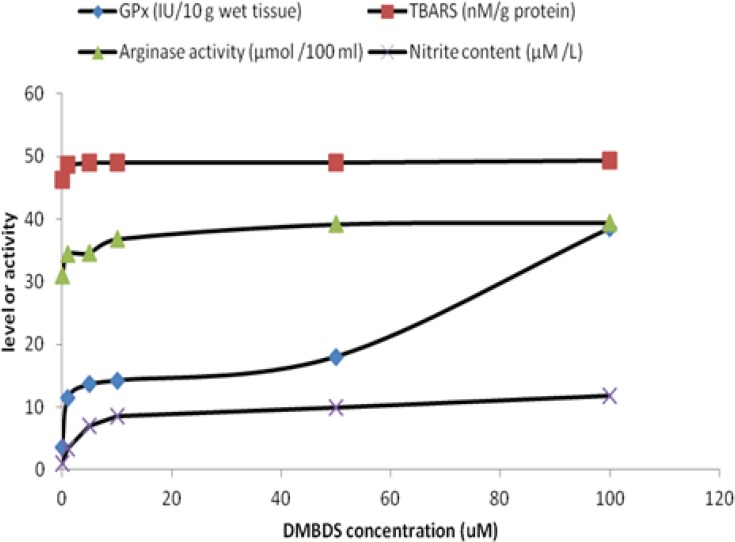
*In-vitro *effect of DMBDS different concentrations on GPx and arginase activities and levels of TBARS and nitrite

Figure 1 showed that GPx and arginase activities as well as TBARS and nitrite levels were linearly increased as DMBDS concentration increased up to 10 μM, while as DMBDS concentration increased from 10 to 100 μM, the activities and levels showed slightly positive increment or zero order curve, at p < 0.05.

**Table 1:** Properties of DMBDS

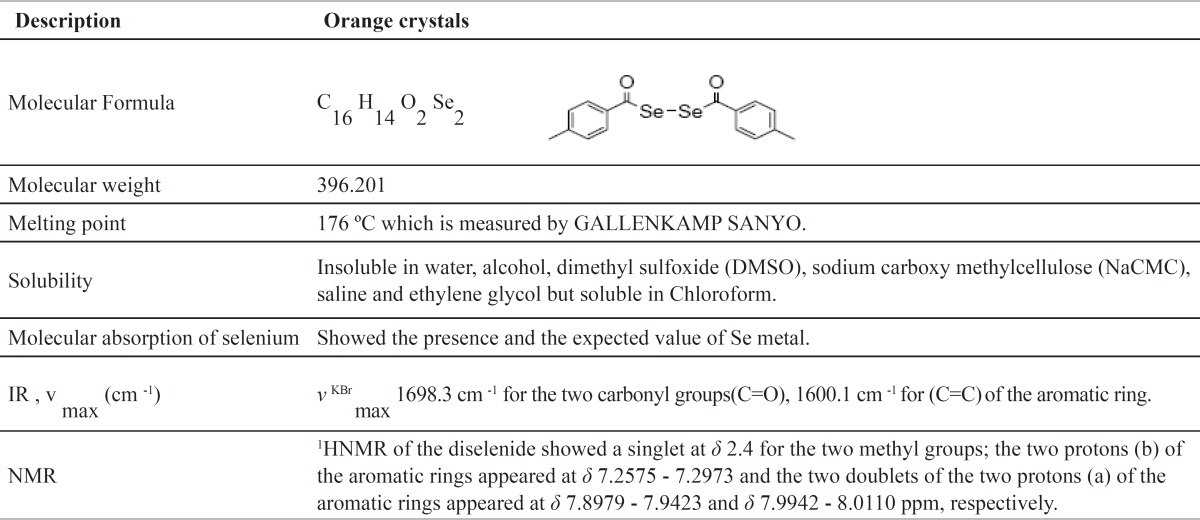

## Discussion

Several studies showed that organoseleno-compounds could be used as antioxidants, enzyme inhibitors, neuroprotectors, antitumor, anti-infectious agents, cytokine inducers and immunomodulators (4, 11, 24). 

The present study was undertaken to evaluate the biological effect of synthesized DMBDS and to estimate its LD50. The obtained results in table (I) supported the structures and the purity of DMBDS through IR, selenium atomic absorption spectra and NMR studies, as confirmed by the reported data carried out by Cho *et al*. (11).

Administration of DMBDS increased GPx activity in direct proportional manner *in-vitro*. That may be attributed to the antioxidant property of selenium incorporated in GPx structure, that result was confirmed by El-Sayed, *et al.*, (25) who stated that selenium containing compounds increased the enzymatic activity through genetic up-regulation. The antioxidant properties of organoselenium compounds have been linked mainly to glutathione peroxidase- or thiol-peroxidase-like activities (i.e., these compounds can decompose peroxides using either reduced glutathione or other thiols). Furthermore, some organoselenium compounds are a substrate for mammalian Trx reductase (TrxR) and can be reduced by electrons derived from NADPH, forming their selenol/selenolate intermediates. These selenol/selenolate intermediates can efficiently decompose hydrogen peroxide (26). Moreover, since 1989 Chada *et al.*, (27) reported that selenium controls human GPx activity and that regulation is exerted at a post-transcriptional level. 

Surprisingly, slight increase in TBARS level concomitant with the elevation of DMBDS that could be attributed to small-inducing effect of examined compound on hepatocytes lipid peroxidation, producing free radicals. This could be returned to the stimulation of detoxification phase II in which, free radicals- glutathione conjugation take place to prevent cellular damage. Furthermore, it was known that nucleophiles antioxidant (such as organoseleno-compound like DMBDS) was better than the classical antioxidants. This concept was encouraged the scientists to design a simple synthetic organoselenium compounds as reported by Meotti *et al., *(10) who found that the alkyl- and aryl-diselenides are antioxidants and the substitution on the aromatic moiety of diphenyldiselenide change the antioxidant property.

Moreover, DMBDS increased NO level which estimated as nitrite content as represented in Figure 1, indicating that DMBDS works also as prooxidant, inducing nitric oxide synthase which oxidizes the guanidine group of L-arginine in a process that consumes five electrons and results in the formation of NO (28). On the other hand, L-arginine is metabolized by several pathways including intracellular arginase, the final enzyme in urea cycle (29). By competing for a common substrate, arginase may affect NO production. Our results showed that DMBDS activated arginase *in-vitro*, which could be attributed to the moderate effect of the examined compound on hepatocytes in order to control the production of NO to prevent the inflammation that could take place in the presence of high level of NO. Also, DMBDS could activate urea cycle which in turn enhances the arginase activity. That result was coincide with Prins, *et al. *(30), who emphasized that arginase activity increased in several cases such as liver cell damage associated with kupffer cells or urea cycle activation. 

Finally, our *in-vitro *data indicated that DMBDS was coagulant compound as it decreased APTT and PT as shown in Table 2 that give indication about the adverse effect of DMBDS on heart tissue and vascular system as it increased the incidence of blood clotting. Finally, this compound is safe because the LD50 in g/Kg body weight.

**Table 2:** *In-vitro *effect of DMBDS on human APTT and PT

**Compound I concentration (mg)**	**APTT (sec.)**	**PT (sec.)**
0	68.50 ± 3.08	35 ± 0.63
0.5	42.83 ± 2.48*	28 ± 1.26*
1.1	42.83 ± 2.64*	28 ± 1.55*

APTT: activated partial thrombin time.

PT: Prothrombin time.

* Significantly different with control-value at *p*< 0.05\

## Conclusion

DMBDS is safe compounds with antioxidants, anti- haemolytic and anti-inflammatory properties but it could be have hepatotoxicity character. On the other hand, this compound is safe as the LD50 in g/Kg but the side effects and toxicity must be done to find the lowest concentration that will produce the hepatotoxicity. 

*Conflict of interest statement*

We declare that there is no disclose any financial and personal relationships with other people or organizations that could inappropriately influence (bias) our work.

## References

[1] Rayman M (2000). The importance of selenium to human health. Lancet.

[2] Spallholz J (1994). On the nature of selenium toxicity and carcinostatic activity. Free Radic. Biol. Med.

[3] Wang H, Zhang J, Yu H (2007). Elemental selenium at nano size possesses lower toxicity without compromising the fundamental effect on selenoenzymes: Comparison with selenomethionine in mice. Free Radic. Biol. Med.

[4] Nogueira C, Zeni G, Rocha J (2001). organoselenium and organotellurium compounds: toxicology and pharmacology. Chem. Rev.

[5] Li Y, Cao Z (2002). The neuroprotectant ebselen inhibits oxidative DNA damage induced by dopamine in the presence of copper ions. Neurosci. Lett.

[6] Yang C, Liu J, Shen H, Ong C (2000). Protective effect of ebselen on aflatoxin B1- induced cytotoxicity in primary rat hepatocytes. Pharmacol. Toxicol.

[7] Farina M, Barbosa N, Nogueira C, Folmer V, Andrade L, Zeni G, Bolzan R, Braga A, Rocha J (2002). Reaction of dipheyl diselenide with hydrogen peroxide and inhibition of delta-aminolevulinate dehydratase from rat liver and cucumber leaves. Braz. J. Med. Biol. Res.

[8] Rosa R, Sulzbacher K, Picada J, Roesler R, Saffi J, Brendel M, Henriques J (2004). Genotoxicity of diphenyl diselenide in bacteria and yeast. Mutat. Res.

[9] Klotz L, Sies H (2003). Defenses against peroxynitrite: selenocompounds and flavonoids. Toxicol. Lett.

[10] Meotti F, Stangherlin E, Zeni G, Nogueira C, Rocha J (2004). Protective role of aryl and alkyl diselenides on lipid peroxidation. Environ. Res.

[11] Cho S, Koketsu M, Ishihara H, Matsushita M, Nairn A, Fukazawa H, Uehara Y (2002). Novel compounds 1;3-selenazine derivatives’ as specific inhibitors of eukaryotic elongation factor-2 kinase. Biochimica. Et. Biophysica. Acta.

[12] Lin H, Tseng H, Wang C, Lin J, Lo C, Chou F (2008). Hepatoprotective effects of Solanum nigrum Linn extract against CCL4-induced damage in rats. Chemico-Biologic. Interact.

[13] Montgomery H, Dymock J (1964). The determination of nitrite in water. Analyst.

[14] Paglia E, Valentine N (1976). Studies on the quantitative and qualitative characterization of erythrocyte glutathione peroxidase. J. Laboratory Clin. Med.

[15] Marsch W (1965). Determination of arginase activity. Clin. Chem.

[16] Wills E (1965). Mechanism of lipid peroxide formation in animals. Biochem. J.

[17] Athukorala Y, Lee K, Kim S, Jeon Y (2007). Anticoagulant activity of marine green and brown algae collected from Jeju island in Korea. Bioresource Technol.

[18] Habig H, Pabst G, Jakoby B (1974). Glutathione-S-transferase The first enzymatic step in mercapturic acid formation.. J. Biol. Chem.

[19] Bardawill C, David M (1949). A colorimetric method for the determination of total protein concentration. J. Biol. Chem.

[20] Reitman S, Frankel S (1957). A colorimetric method for the determination of serum glutamic oxaloacetic and glutamic pyretic tranaminases. Am. J. Clin. Path.

[21] Larsen K (1972). A colorimetric kinetic method for the determination of creatinine. Clin. Chem. Acta.

[22] Fawcett J, Scott J (1960). Determination of urea. J. Clin. Path.

[23] Tietz N (1990). Clinical guide to laboratory tests.

[24] Chen T, Wong Y, Zheng W, Liu J (2009). Caspase- and p53-dependent apoptosis in breast carcinoma cells induced by a synthetic selenadiazole derivative. C.B.I.

[25] EL-Sayed W, Aboul-Fadl T, Lamb T, Roberts J, Franklin M (2006). Effect of selenium-containing compounds on hepatic chemoprotective enzymes in mice. Toxicol.

[26] Freitas A, Prestes A, Wagner C, Sudati J, Alves D, Porciúncula L, Kade I, Rocha J (2010). Reduction of diphenyl diselenide and analogs by mammalian thioredoxin reductase is independent of their gluthathione peroxidase-like activity: a possible novel pathway for their antioxidant activity. Molecules.

[27] Chada S, Whitney C, Newburger P (1989). Post-transcriptional regulation of glutathione peroxidase gene expression by selenium in the HL-60 human myeloid cell line. Blood.

[28] Ignarro L (1990). Nitric Oxide, A Novel Signal Transduction Mechanism For Transcellular Communication. Hyperten.

[29] Zharikov S, Krotova K, Baylis C, Johson R, Patel J (2008). Uric acid decreases NO production and increases arginase activity in cultured pulmonary artery endothelial cells. J. Am. J. Physiol. Cell Physiol.

[30] Prins H, Meijer C, Nijveldt R, Wiezer M (2000). High plasma levels of arginine and liver arginase in Kupffer-cell-depleted rats after partial hepatectomy. J. Hepatol.

